# Racial and socioeconomic inequalities in the financial stress of medical school

**DOI:** 10.12688/mep.17544.1

**Published:** 2022-01-19

**Authors:** Brennan McMichael, Anderson Lee IV, Brian Fallon, Niki Matusko, Gurjit Sandhu

**Affiliations:** 1 Department of Surgery, University of Michigan, Ann Arbor, MI, 48109, USA; 2 Department of Anesthesia, University of Michigan, Ann Arbor, MI, 48109, USA

**Keywords:** Medical student debt, educational debt burden, underrepresented in medicine (URiM), financial literacy, wellness

## Abstract

**Background:** The authors analyzed the distribution of medical student debt and identified demographic features that placed students at high risk for increased debt and financial stress.

**Methods: **From April to May 2019, a cross-sectional, anonymous, web-based survey was administered to first-year (M1) to fourth-year (M4) medical students at the University of Michigan to assess financial literacy, debt burden, financial stress, and demographic factors. A total of 216 of 680 (32%) students completed the survey. Respondents voluntarily answered 15 multiple-choice questions on personal finance and 30 questions on their demographics, current financial situation, and debt burden. To quantify debt burden, students estimated anticipated education-related debt in one of four categories: no debt, $1–99,999; $100,000–$199,999; and $200,000 or more. A chi-square test was used to identify associations between categorical variables and logistic regression was used to identify risk factors for debt and worry.

**Results: **Fifty-four respondents (25%) reported $0 in education related debt, while 44 (16%) had $200,000 or more. Race (p=0.006), first-generation college student status (p=0.004), first-generation medical student status (p<0.001), household income (p<0.001), and parental education (p=0.008) were associated with higher levels of debt. Students who were underrepresented in medicine (URiM) had higher odds of higher debt compared to Arab and Asian students (p=0.02). URiM students (p=0.02), first-generation college students (p=0.009), and parental education (p=0.01) were associated with increased financial stress. Additionally, female students had higher odds of increased financial stress (OR=1.85, p=0.045) on logistic regression.

**Conclusions:** URiM and socioeconomically disadvantaged students feel the burden of the high cost of medical school disproportionately more, suggesting that our current systems are not adequately supporting these students. Reducing this burden may serve to further promote diversity in medicine.

## Introduction

The cost of obtaining a medical education exceeds the budget of nearly all students who attend, with the majority of students graduating in debt, and their median indebtedness amounting to an alarming $200,000 ^1^. Moreover, for students belonging to a group considered underrepresented in medicine (URiM) by the Association of American Medical Colleges (AAMC) ^2^, average debt can rise by as much as $30,000 ^1^. Studies indicate cost to be a principal deterrent for potential URiM and socioeconomically disadvantaged individuals applying to medical school, undermining increased efforts to diversify the medical profession and address disparities in health care ^3, 4^. This inequity seems to be well appreciated, as students from URiM groups receive scholarship funds at a higher rate than the national average. Yet, despite access to scholarships, URiM students still require more loans and receive less financial support from those close to them ^1^. To address this debt gap, many schools have implemented pipeline programs to provide greater financial, equipment, and academic support ^4^, while a smaller number of programs have crafted their own more progressive commitments to reducing medical student debt through debt-free or tuition free initiatives in order to help diversify medical school enrollment ^5, 6^.

Debt not only deters students from entering the medical profession, but also has implications for choice of residency and area of practice. Students may eventually pay more than two times their original loan amount when accounting for accruing interest, consequentially deterring these students from entering less lucrative primary care specialties like pediatrics and family medicine ^7, 8^. Moreover, Pisaniello *et al.* not only found large medical school debt burdens to be associated with final specialty choice, but also academic performance and financial stress ^9^. For residents, finance related concerns, such as increased debt, have been correlated with decreased satisfaction with training and increased rates of burnout ^10– 12^. These financial disparities likely translate to disparities in academic and psychological stress. However, current literature investigating inequality related to medical-student loans is sparce. While racial disparities exist in loan burden, there is a poor understanding of the factors that put medical students at increased risk for large debt burdens, as well as their ensuing downstream effects.

While there have been national policy discussions addressing mounting undergraduate student debt, there have been relatively few that target the medical student debt burden. Further exploration of the intersection of medical-student loans, racial disparities, and financial stress and wellbeing may lend a greater voice to the subject, and ultimately contribute to the subject’s representation and discussion at higher organizational levels, where systematic changes may be enacted. The purpose of this study was to assess distribution of medical student debt and prevalence of financial stress among medical students, as well as identify demographic features that place students at high risk for increased debt and financial stress.

## Methods

### Study participants

A cross-sectional, anonymous, 51-item survey ^13^ that queried recipients’ demographic data, financial literacy level, current debt level, concern about current financial status, and difficulty paying bills was distributed in April 2019 to all medical students (n=680) at the University of Michigan Medical School. The voluntary survey was administered using Qualtrics (Qualtrics, Provo, UT) and invitations were sent via email. To incentivize completion of the survey, four $50 gift cards were awarded to participants in a random drawing. Participation in the incentive was optional and was not linked to survey responses.

### Measures

The survey content was based on a review of the literature ^14– 16^, and included a financial literacy assessment portion that was adapted from the validated Financial Industry Regulatory Authority (FINRA) Financial Literacy Quiz ^17^. To quantify debt burden, students estimated anticipated education-related debt in one of four categories: no debt, $1–99,999; $100,000–$199,999; and $200,000 or more. Financial stress was assessed with the question “Do you worry about your current financial status?”, with three possible responses: “not concerned”, “somewhat concerned”, and “very concerned”. The racial and ethnic populations considered “underrepresented in medicine” (URiM) were based on the definition used by the AAMC: “those racial and ethnic populations that are underrepresented in the medical profession relative to their numbers in the general population ^2^.” The options included in this survey were American Indian or Alaskan Native, Black or African American, Latino, and Native Hawaiian or Pacific Islander. Prior to administration to the entire cohort, the survey was administered to a group of four medical students whose feedback was utilized to make changes to the survey. This study was approved by the University of Michigan Institutional Review Board, and informed consent was obtained from all participants for participation in, and publication of, this study. 

### Statistical analysis

Sample characteristics are described as number of observations and percent for categorical variables and mean and standard deviation for continuous variables. Primary analysis of the data examining the relationship between financial literacy scores, interest in taking a financial literacy course, and the perceived importance of increasing financial literacy has been previously reported (manuscript under review). Chi-square/Fisher’s exact tests were used to identify associations between categorical variables. Multivariable ordered logistic regression analysis was used to test the association between potential demographic risk factors and increasing debt and worry. Multivariable logistic regression was used to assess the relationship between identified factors and the ability to cover monthly expenses and worry. Separate Chi-square/Fisher’s exact tests were used to ascertain the association between race/ethnicity and the primary outcomes among the first-generation medical students subgroup. All analyses were conducted in STATA 15 (StataCorp, 2017) and significance was set at p<0.05.

## Results

This study on medical student debt and financial worry distributed surveys to 680 medical students at the University of Michigan Medical School. Overall, 216 respondents completed the survey (31%). There were 32 selections among all responses identifying with a racial or ethnic group considered URiM ( Table 1). Multiple students identified with more than one racial or ethnic group. Our sample contained 130 (60%) females and 85 (39%) males. Respondents were further categorized according to their year in school as M1/M2 (n=123, 57%) and M3/M4 (n=91, 43%). There were 36 (17%) respondents who were first-generation college students and 155 (73%) that were first-generation medical students. There was no significant difference between the proportion of first-generation college students and the proportion of first-generation medical students across all races and ethnicities. There were 99 (49%) students that came from households with an annual household income of $75,000 or greater. A smaller proportion of URiM students came from a household with annual incomes of $75,000 or greater.

**Table 1.** Demographic Characteristics of Medical Student Participants.

Demographic Factors	# of Participants in the Study (N=216)
**Age (years)**	
21 – 24	51 (24%)
25 – 28	122 (57%)
29 – 32	35 (16%)
>33	7 (3%)
**Sex**	
Male	85 (39%)
Female	130 (60%)
Other	1 (1%)
**Race/ethnicity**	
American Indian or Alaskan Native *	3 (1%)
Arab	5 (2%)
Asian	43 (18%)
Black or African American *	19 (8%)
Latino *	9 (4%)
Native Hawaiian or Pacific Islander *	1 (0%)
White	150 (64%)
Other	5 (2%)
**Medical school year**	
M1 - M2	91 (43%)
M3 - M4	123 (57%)
**Annual household income**	
<$20,000	46 (23%)
$20,000 - $34,999	23 (12%)
$35,000 - $49,999	13 (7%)
$50,000 - $74,999	19 (9%)
$75,000 - $99,999	16 (8%)
>$100,000	83 (41%)
**First-generation college student**	
Yes	36 (17%)
No	177 (83%)
**First-generation medical student**	
Yes	155 (73%)
No	58 (27%)
**Expected medical loan debt at graduation**	
$0	54 (25%)
$1 - $99,999	45 (21%)
$100,000 - $199,999	68 (31%)
>$200,000	44 (20%)
I don't know	5 (2%)

*Denotes a group considered under-represented in medicine by the Association of American Medical Colleges.

### Debt burden

Our sample contained 54 (25%) medical students with no anticipated education related debt; 45 (21%) with $1–99,999; 68 (32%) with $100,000–$199,999; and 44 (16%) with $200,000 or more anticipated education related debt. Factors that were found to be significantly associated with higher levels of debt were race (p=0.006), status as a first-generation college student (p=0.004), status as a first-generation medical student (p<0.001), household income (p<0.001), and parental education (p=0.008). Age was not found to be significantly associated with education-related debt. On multivariable ordered logistic regression, Arab and Asian students were at lower odds of being in higher debt categories when compared to URiM students (OR = 0.35, p=0.02). There was no significant difference between URiM students and white students. First-generation medical students were at higher odds of being in high debt categories than those students who were not first-generation medical students (OR=2.20, p=0.02).

### Financial stress

Most medical students (178; 82%) are either very or somewhat concerned about their current financial status ( Table 2). On multivariable ordered logistic regression, Arab and Asian students were at lower odds of being in the higher concern categories when compared to URiM students (OR =0.30, p=0.02). Additionally, female medical students (OR=1.85, p=0.045) had higher odds of being in the higher concern categories than their counterparts ( Table 4). Age and household income were not associated with concern about current financial status. Difficulty covering monthly expenses was reported by 75 (36%) medical students ( Table 3). The factors significantly associated with difficulty related to covering monthly expenses were status as a first-generation college student (p<0.001) and first-generation medical student (p=0.008). However, these significant associations attenuated in the multivariable logistic regression analysis.

**Table 2.** Results of Chi-square and Fisher’s exact tests analysis of demographic characteristics association with medical student financial worry.

Age	Not concerned	Somewhat concerned	Very concerned	P-Value
21–24	6 (11.76)	36 (70.59)	9 (17.65)	0.266
25–28	21 (17.21)	72 (59.02)	29 (23.77)	
29–32	10 (23.81)	20 (47.62)	12 (28.57)	
**Race**				
white	22 (16.79)	79 (60.31)	30 (22.90)	0.024
URiM	1 (3.33)	17 (56.67)	12 (40.00)	
Arab or Asian	13 (27.08)	28 (58.33)	7 (14. 58)	
**First-generation college student**				
Yes	3 (8.33)	18 (50.00)	15 (41.67)	0.009
No	35 (19.77)	108 (61.02)	34 (19.21)	
**First-generation medical student**				
Yes	22 (14.19)	93 (60.00)	40 (25.81)	0.072
No	15 (25.86)	34 (58.62)	9 (15.52)	
**Annual Household Income**				
75K+	12 (11.88)	68 (67.33)	21 (20.79)	0.169
<75K	20 (20.20)	55 (55.56)	24 (24.24)	
**Parental Education**				
College Graduate	34 (19.10)	110 (61.80)	34 (19.10)	0.011
Other	3 (9.09)	16 (48.48)	14 (42.42)	
**Gender**				
Male	18 (21.18)	54 (63.53)	13 (15.29)	0.071
Female	20 (15.38)	73 (56.15)	37 (28.46)	

Note: URiM= underrepresented in medicine. The number of responses is listed first, followed by percentage of responses in parenthesis.

**Table 3.** Results of Chi-square and Fisher’s exact tests analysis of demographic characteristics association with medical student expenses.

Age	Difficulty covering monthly expenses	No difficulty covering monthly expenses	P-Value
21–24	34 (66.67)	17 (33.33)	0.067
25–28	85(69.67)	37 (30.33)	
29–32	21 (50.00)	21 (50.00)	
**Race**			
white	82 (62.60)	49 (37.40)	0.128
URiM	16 (53.33)	14 (46. 67)	
Arab or Asian	36 (75.00)	12 (25.00)	
**First-generation college student**			
Yes	14 (38.89)	22 (61.11)	0.000
No	124 (70.06)	53 (29.94)	
**First-generation medical student**			
Yes	93 (60.00)	62 (40.00)	0.008
No	46 (79.31)	12 (20.69)	
**Annual Household Income**			
75K+	60 (59.41)	41 (40.59)	0.288
<75K	66 (66.67)	33 (33.33)	
**Parental Education**			
College Graduate	124 (69.66)	54 (30.34)	0.001
Other	13 (39.39)	20 (60.61)	
**Gender**			
Male	59 (69.41)	26 (30.59)	0.285
Female	81 (62.31)	49 (37.69)	

Note: URiM= underrepresented in medicine. The number of responses is listed first, followed by percentage of responses in parenthesis.

**Table 4.** Results of multivariable ordered logistic regression analysis of demographic parameters contributing to medical student financial worry.

	OR	95.0% CI	P-vale
**Age**			
21–24	Reference	-	-
25–28	1.24	0.617-2.504	0.543
29–32	1.06	0.431-2.59	0.905
**Race**			
URiM	Reference	-	-
white	0.54	0.231-1.243	0.146
Arab or Asian	0.30	0.112-0.820	0.019
First Generation College Student (Yes)	0.30	0.032-2.701	0.279
First Generation Medical Student (Yes)	1.84	0.913-3.715	0.088
Annual Household Income (75K+)	1.10	0.605-2.006	0.752
Parental Education (college graduate)	0.13	0.014-1.286	0.082
Gender (female)	1.85	1.014-3.390	0.045

*Note: OR = odds ratio, CI = confidence interval

### Subgroup analysis: first-generation medical students

There were 149 respondents who identified as first-generation medical students. No statistically significant differences were found with regards to student debt or financial stress when first-generation medical students who were URiMs were evaluated against their medical school counterparts. There were 26 (17%) first-generation medical students that identified as URiM; nearly all (n=25, 97%) responded that they were “somewhat concerned” or “very concerned” about their current financial status ( Table 2).

## Discussion

This single institution survey of medical students found that most students will graduate with significant student debt and financial stress. Our results are consistent with national data regarding medical student debt and relative disparities in debt ^1^. Our findings indicate that medical students display a high level of finance-related stress related to their current financial status. There were four factors that placed students at high risk for financial stress: identifying as URiM, first-generation college student, first-generation medical student, and female. Additionally, there is potential for these factors to work synergistically when added together, further compounding financial debt and stress, as many of our students belonged to more than one of these at-risk groups. For example, being a first-generation college or medical student was associated with higher debt, but URiM students were just as likely as other racial or ethnic groups to be a first-generation college or medical student. Additionally, URiM students were less likely to indicate a high family income—which is a protective factor against high debt. This data can serve to enhance the current discourse regarding an important impediment to increasing health care diversity and lead to the development of interventions to combat medical student financial stress.

The finding that race, status as a first-generation college student, status as a first-generation medical student, household income, and parental education were associated with higher debt emphasizes the need to further explore the extent of the impact of this barrier to medical school diversification. While the AAMC has encouraged medical schools to utilize holistic reviews that account for life experiences, cultural competence, and other attributes to promote more diverse student populations ^18^, additional progressive initiatives are necessary. Recently, Thomas *et al.* found that, while the overall number of applicants to US medical schools increased by 47% from 1980 to 2016, the numbers of Black or African American and Hispanic or Latino applicants increased by only 1.2%, and the numbers of Alaska Native and American Indian applicants declined by 29% ^19^. Additionally, the literature shows that first-generation college students encounter challenges negotiating the college process, financing their education, and balancing education with commitments at home ^20, 21^. As such, our results have potential implications for the recruitment and retention of medical students from these backgrounds.

Medical students who identified as URiM, female, a first-generation college student, or a first-generation medical student are under increased financial stress, as evidenced by their higher level of financial concern, and for the latter two groups, difficulty paying bills. A recent systematic review examined the effect of medical student debt on mental health, finding financial stress was typically associated with debt levels, troubling patterns of alcohol use, and adverse effects on academic performance ^9^. Our examination of the distribution of this well-described, debt-related stress is vital to supporting more at risk students and creating targeted systematic approaches to increasing access and representation to these groups in the medical field going forward. Nationally, students from these backgrounds receive scholarships at a higher rate than average, indicating that the medical education system is aware of the unique challenges that these students face ^1^. Yet, our results indicate that they are still at high-risk for financial stress despite additional scholarships.

While loans are important for students to pay the cost of their education, it is incumbent on medical curriculums to equip students with the personal finance knowledge to be able to effectively manage their debt burden. Many financial literacy programs have been targeted at residents ^22– 24^, but this is evidently too late, allowing for harm to mental health, hindering academic success, and affecting pivotal decisions during medical school years ^7, 9, 25^. Medical school programs should look to engage students to develop financial literacy education and integrate self-directed learning opportunities, provide mental health support regarding finances, and explore viable ways to address the cost of this education for students from socioeconomically disadvantaged backgrounds.

To our knowledge, there are no previously published studies exploring the distribution of medical student debt and demographic features that place students at risk for increased debt and financial stress. As a pilot study to better understand medical student debt and stress, there were several limitations to our study. We conducted our study at a single institution, which may prevent results from being generalized to the medical student population nationally. A second limitation was that a convenience sampling method was used. This also limits the ability to generalize these results. Lastly, the sample size of this study was small and would benefit from dissemination to a national medical student body.

In conclusion, this study shows URiM and socioeconomically disadvantaged students feel the burden of the high cost of medical school disproportionately more than their counterparts. This suggests that our current systems are not adequately supporting these students. We hope the findings from this study will contribute to important discourse regarding systemic approaches and changes to reducing this burden, and the subsequent impact on wellbeing, that may serve to further promote diversity in medicine.

## Data availability

### Underlying data

Under the IRB for this project, data is only to be accessed by the PI and co-investigators. To access data, individuals would need to ask for approval from the University of Michigan (UM) IRB. Any shared data would be completely deidentified to ensure there is not the possibility of matching survey data to individual medical students and data would only be available for further research purposes. Upon approval by the UM IRB, the corresponding author, Gurjit Sandhu, would share the data with the requestor. To contact the UM IRB, email **irbmed@umich.edu**.

### Extended data

Deep Blue Documents: Financial Literacy Survey Instrument. https://dx.doi.org/10.7302/3727 ^13^

This project contains the following extended data:

- Financial Literacy Survey Instrument.docx (Financial Literacy Survey Instrument)

Data are available under the terms of the Creative Commons Attribution 4.0 International license (CC-BY 4.0).
