# Supplementary material for: Racial and socioeconomic inequity in the financial stress of medical school
Source: MedEdPublish (2016). 2022 Jun 13;12:3. Originally published 2022 Jan 19. [Version 2] doi: 10.12688/mep.17544.2 (PMC9370082; doi:10.12688/mep.17544.2)
Supplement: Peer review history

# Peer review: Reviewer response for version 2

**DOI:** 10.21956/mep.20555.r32127

**Published:** 2022-06-15

**Authors:** Phillippa Poole

**Affiliations:** 1 School of Medicine, University of Auckland, Auckland, New Zealand

I am happy to approve this version.

Have any limitations of the research been acknowledged?

Yes

Is the work clearly and accurately presented and does it cite the current literature?

Yes

If applicable, is the statistical analysis and its interpretation appropriate?

Yes

Are all the source data underlying the results available to ensure full reproducibility?

Yes

Is the study design appropriate and is the work technically sound?

Yes

Are the conclusions drawn adequately supported by the results?

Yes

Are sufficient details of methods and analysis provided to allow replication by others?

Yes

Reviewer Expertise:

NA

I confirm that I have read this submission and believe that I have an appropriate level of expertise to confirm that it is of an acceptable scientific standard.

# Peer review: Reviewer response for version 2

**DOI:** 10.21956/mep.20555.r32126

**Published:** 2022-06-14

**Authors:** Jamiu Busari

**Affiliations:** 1 Educational Development and Research Department, Faculty of Health, Medicine and Life Sciences, Maastricht University, Maastricht, The Netherlands; 2 Dept of Pediatrics, Horacio Oduber Hospital, Oranjestad, Aruba

The authors have satisfactorily addressed the concerns raised regarding the previous version of this manuscript.

 With the attention given to pre-existent inequities among URiM students and how these inequities influence the students' admission to medical school and subsequently contribute to study attrition and debt accrual, the authors provide a balanced description of the findings in relation to the research problem. Therefore, I am happy to recommend acceptance of this revised manuscript. Congratulations on this valuable addition to the literature.

Have any limitations of the research been acknowledged?

Yes

Is the work clearly and accurately presented and does it cite the current literature?

Yes

If applicable, is the statistical analysis and its interpretation appropriate?

Yes

Are all the source data underlying the results available to ensure full reproducibility?

Yes

Is the study design appropriate and is the work technically sound?

Yes

Are the conclusions drawn adequately supported by the results?

Yes

Are sufficient details of methods and analysis provided to allow replication by others?

Yes

Reviewer Expertise:

Leadership in medical education and practice, Faculty development, DEI in health systems and education

I confirm that I have read this submission and believe that I have an appropriate level of expertise to confirm that it is of an acceptable scientific standard.

# Peer review: Reviewer response for version 1

**DOI:** 10.21956/mep.18819.r31995

**Published:** 2022-05-31

**Authors:** Phillippa Poole

**Affiliations:** 1 School of Medicine, University of Auckland, Auckland, New Zealand

Important topic. Paper extremely well-written and argued and adds to the understanding of the factors contributing to financial stress, and indeed participation in medical education in the first place. Agree that there is little in the literature on this topic.

 The authors may like to consider the following:

- In the conclusion, it is stated this is a pilot study. It might be worth indicating earlier in the aim that is exploratory.      
- Methods – student survey with self-identified demographic characteristics. Population 690, but only 31% response rate. Only 32 URIM. Can this be discussed more as to how this may have affected the results?
- It might be clearer why you surveyed students in both early and later years of the programme. Did you do a comparison between the two? There may be changes in the levels of stress over time as well as different factors in this.
- Would you explain further how you chose the variables for the multivariable ordered logistic regression analysis to test the association between potential demographic risk factors and increasing debt and worry? How much variance in the outcome did the model explain?
- Small numbers in cells are an issue: they may introduce bias, plus was care taken to reduce the chance of identification of individuals who had answered the survey?   
- An implicit aim of the paper is to increase the participation of underrepresented groups, so terminology is important to support diversity and convey respect. See for example Annette Flanagin, RN, MA; Tracy Frey, BA; Stacy L. Christiansen, MA; for the AMA Manual of Style Committee. Updated Guidance on the Reporting of Race and Ethnicity in Medical and Science Journals. JAMA 2021;326 (7):621-627. ^1^
  Suggest considering: male/female refers to biological sex. Gender terms would be men, women, non-binary.  
  Use of terms race vs. ethnicity vs ancestry.
- As a general comment to the journal team: the need to scroll in the tables distracted considerably from my appreciation of this work.  

Thank you for the opportunity to review.

Have any limitations of the research been acknowledged?

Yes

Is the work clearly and accurately presented and does it cite the current literature?

Yes

If applicable, is the statistical analysis and its interpretation appropriate?

Yes

Are all the source data underlying the results available to ensure full reproducibility?

Yes

Is the study design appropriate and is the work technically sound?

Yes

Are the conclusions drawn adequately supported by the results?

Yes

Are sufficient details of methods and analysis provided to allow replication by others?

Yes

Reviewer Expertise:

NA

I confirm that I have read this submission and believe that I have an appropriate level of expertise to confirm that it is of an acceptable scientific standard.

## References

[1] : Updated Guidance on the Reporting of Race and Ethnicity in Medical and Science Journals. *JAMA* .2021;326(7) : 10.1001/jama.2021.13304 10.1001/jama.2021.13304 34402850

# Peer review: Reviewer response for version 1

**DOI:** 10.21956/mep.18819.r31946

**Published:** 2022-05-25

**Authors:** Jamiu Busari

**Affiliations:** 1 Educational Development and Research Department, Faculty of Health, Medicine and Life Sciences, Maastricht University, Maastricht, The Netherlands; 2 Dept of Pediatrics, Horacio Oduber Hospital, Oranjestad, Aruba

This interesting study sheds light on the financial burden of medical education in URiM. Furthermore, it highlights the impact of inequalities attributed to students' race and socioeconomic status in medical school.

 The study does not add anything new for the "versed" observer about what is already known about the subject or what could be expected from the research question. it does however offer the required proof (represented in data) that can help justify the discourse on this subject.

 The introduction is informative and provides a good background of the problem. Yet, I believe that the content provided in this paper can be tightened up into two paragraphs by the authors. Also the methods design and chosen intervention seem credible and achieved a 30% response rate which is commendable for an online survey.

 The analysis and interpretation of data are acceptable, and the content of the discussion aligns well with the study's findings. My major critiques of the paper are twofold:

 First, the authors present the case for this paper as racial and socioeconomic inequalities. However, in my opinion, the problem described in this study goes beyond inequality. It should (also) be about inequity which should be explicitly described as an issue at hand. The higher rate of a lack of access to funds for training and the inability to repay because of their parents' socioeconomic background or level of education are preexistent factors that do not necessarily amount to inequality but determine the distribution of opportunity and privileges. These factors are not considered as entry into medical programs assumes that all students are entering from the same starting point. So, I firmly believe that the authors need to emphasize the role ()equity plays in this process, which they have implicitly touched on in the manuscript.

 My second critique is that the authors did not mention the impact of the "minority tax" on URiM students and its role in high debt accrual, study attrition, and financial stress. Also, how this contributes to the leaky pipeline phenomenon in medical education needs to be discussed. 

 While I acknowledge the importance of this study, the relevance of the findings needs to be reframed.  They should also be positioned differently within equity in medical training. Finally, this manuscript needs to offer more than just reporting what many already know or experience in real life, especially for marginalized readers.

 I commend the authors for their interesting work.

Have any limitations of the research been acknowledged?

Yes

Is the work clearly and accurately presented and does it cite the current literature?

Yes

If applicable, is the statistical analysis and its interpretation appropriate?

Yes

Are all the source data underlying the results available to ensure full reproducibility?

Yes

Is the study design appropriate and is the work technically sound?

Yes

Are the conclusions drawn adequately supported by the results?

Yes

Are sufficient details of methods and analysis provided to allow replication by others?

Yes

Reviewer Expertise:

Leadership in medical education and practice, Faculty development, DEI in health systems and education

I confirm that I have read this submission and believe that I have an appropriate level of expertise to confirm that it is of an acceptable scientific standard, however I have significant reservations, as outlined above.

# Peer review: Reviewer response for version 1

**DOI:** 10.21956/mep.18819.r31577

**Published:** 2022-04-04

**Authors:** Rashmi A. Kusurkar

**Affiliations:** 1 Amsterdam UMC, Vrije Universiteit Amsterdam, Faculty of Medicine, De Boelelaan 1118, 1081HZ, The Netherlands; 2 LEARN! Research Institute for Learning and Education, Faculty of Psychology and Education, VU University Amsterdam, Amsterdam, The Netherlands

This article investigates financial study-related debt and worries that medical students have during or at the end of their medical study. It explores if all students perceive similar difficulties or do certain groups like URiM students perceive greater difficulties. 

 The study is designed and executed well. The analysis and its interpretation is done well. The conclusions are parsimonious. What I would have liked to see reported is the finance literacy scores of the students and how the demographic factors are related to them. I recommend to the authors to add this to the results. After all they have collected this data.

Have any limitations of the research been acknowledged?

Yes

Is the work clearly and accurately presented and does it cite the current literature?

Yes

If applicable, is the statistical analysis and its interpretation appropriate?

Yes

Are all the source data underlying the results available to ensure full reproducibility?

Yes

Is the study design appropriate and is the work technically sound?

Yes

Are the conclusions drawn adequately supported by the results?

Yes

Are sufficient details of methods and analysis provided to allow replication by others?

Yes

Reviewer Expertise:

Diversity; motivation; learning; professional

I confirm that I have read this submission and believe that I have an appropriate level of expertise to confirm that it is of an acceptable scientific standard.
